# Clinical experience of transumbilical single-port laparoscopic cholecystectomy using a modified glove-port device and conventional instruments

**DOI:** 10.1186/s12893-026-04102-9

**Published:** 2026-08-01

**Authors:** Shuming Xiong, Jun Yang, Maoqun Zhu, Benshun Hu

**Affiliations:** Department of Hepatobiliary Surgery, The Affiliated Hospital of Jiangnan University, Wuxi, Jiangsu China

**Keywords:** Single-port laparoscopy, Cholecystectomy, Propensity score matching, Custom port, Transumbilical, Conventional instruments

## Abstract

**Background:**

The use of single-port laparoscopic cholecystectomy (SPLC) with disposable multichannel ports is constrained in resource-limited primary hospitals under China’s diagnosis-related groups (DRGs) payment model, where consumable expenditures are systematically controlled. This study reports a practical modification of the glove-port technique using conventional instruments for transumbilical single-port laparoscopic cholecystectomy (TUSPLC) and compares its single-center clinical outcomes with those of conventional three-port LC.

**Methods:**

A retrospective, non-randomized, comparative cohort analysis was performed on consecutive eligible patients who underwent TUSPLC (study group) or traditional three-port LC (control group) between October 2023 and December 2024 at a single center. Baseline demographics, perioperative outcomes, hospitalization costs, and short-term patient-reported cosmetic satisfaction were compared. Propensity score matching (PSM) was performed to adjust for selection bias on age, sex, BMI, ASA classification, and disease type.

**Results:**

A total of 173 patients underwent TUSPLC and 242 patients underwent three-port LC. After nearest-neighbor 1:1 PSM on age, sex, BMI, ASA grade, and gallbladder disease type, 162 pairs were retained for analysis. Matching yielded well-balanced groups (all standardized mean differences below 0.1). Three TUSPLC cases required conversion to multi-port surgery (1.7%) — two because of difficult Calot’s triangle dissection and one for severe gallbladder atrophy. No bile duct injury, postoperative hemorrhage, or surgical site infection was recorded in either group. Operative time (42.0 ± 5.1 vs. 41.2 ± 4.6 min, mean difference 0.80 min, 95% CI -0.3 to 1.9, *p* = 0.140), blood loss (5.9 ± 1.5 vs. 5.7 ± 1.4 mL, mean difference 0.20 mL, 95% CI -0.1 to 0.5, *p* = 0.220), and total cost (14,376.2 ± 982.5 vs. 14,437.3 ± 856.7 CNY, mean difference − 61.1 CNY, 95% CI − 262.6 to 140.4, *p* = 0.551) did not differ significantly between the two approaches (all *p* > 0.05). Postoperative hospital stay was statistically shorter in the TUSPLC group (2.0 ± 0.5 vs. 2.1 ± 0.4 days, mean difference − 0.10 day, 95% CI -0.2 to 0.0, *p* = 0.048), although the absolute difference was small and of uncertain clinical significance. Visual analogue scale (VAS) scores at 1 month favored TUSPLC (9.23 ± 0.45 vs. 8.52 ± 0.68, mean difference 0.71, 95% CI 0.6 to 0.8, *p* < 0.001), although the assessment was unblinded and patient-reported.

**Conclusions:**

TUSPLC using a modified glove-port device and conventional instruments appears technically feasible within the short-term (1-month) follow-up period in selected patients, with no statistically significant differences in operative time, blood loss, or total hospitalization costs compared with conventional three-port LC. Postoperative hospital stay was statistically shorter by 0.10 day, a difference of uncertain clinical significance. This modified technique may serve as a practical option for performing single-port LC in resource-limited primary-level hospitals under China’s DRGs payment model. However, because of the retrospective, single-center design and short (1-month) follow-up, long-term safety data for port-site hernia and other delayed complications are lacking, and the cosmetic benefit requires validation with objective assessments; therefore, these findings should be interpreted as preliminary. Further prospective, multicenter studies with extended follow-up are essential.

Since Mühe [1] first performed a laparoscopic cholecystectomy (LC) in 1985, LC has become the gold standard for treating benign gallbladder diseases [2–4]. With the continuous advancement of LC technology and accumulated clinical expertise, surgical incisions for cholecystectomy have evolved from traditional four-port and three-port techniques to the currently trending single-port laparoscopic cholecystectomy (SPLC) approach [2–6]. The purpose of using the SPLC approach was to reduce surgical trauma and postoperative pain, while meeting patients’ demand for improved cosmetic outcomes [3, 4, 7, 8]. The safety and feasibility of SPLC have been widely established in the medical literature [4, 9–11]. However, under China’s diagnosis-related groups (DRGs) payment model, the substantial cost of disposable single-incision laparoscopic ports limits their widespread clinical adoption [12, 13]. Additionally, when performing single-incision laparoscopic surgery with conventional instruments, frequently encountered issues such as the “chopstick effect” and intraoperative gas leakage impose significant operational inconveniences and challenges on surgeons [2, 8, 14].

Against this background, we modified a previously described glove-port technique [15] to perform transumbilical single-port laparoscopic cholecystectomy (TUSPLC) using conventional instruments. This retrospective, single-center, non-randomized comparative cohort study reports our clinical experience with this modified technique. We compare its perioperative outcomes with conventional three-port LC to assess short-term feasibility, intraoperative safety, hospitalization costs, and 1-month cosmetic satisfaction in resource-limited medical environments.

## Materials and methods

### General information

We conducted a single-center, retrospective, non-randomized cohort study. All consecutive eligible patients who underwent laparoscopic cholecystectomy at our center from October 2023 to December 2024 were retrospectively identified. All patients received standardized preoperative counseling regarding the surgical procedures, potential risks, advantages, and cosmetic outcomes of TUSPLC and three-port LC, and subsequently made independent decisions after providing full informed consent. All operations (both TUSPLC and three-port LC) were performed by 3 senior surgeons in the Department of Hepatobiliary Surgery, all with more than 5 years of laparoscopic surgery experience and having performed over 50 single-port laparoscopic procedures. The first 20 consecutive TUSPLC cases were excluded as the learning curve period based on cumulative sum (CUSUM) analysis of operative time; the mean operative time for these excluded cases was substantially longer than the post-learning-curve cohort, confirming a significant learning-curve effect. The surgical operation process and technical standards were unified to ensure the consistency of surgical level. In particular, acute cholecystitis was diagnosed and graded according to the Tokyo Guidelines 2018 (TG18) [16]. Patients were assigned to either the transumbilical single-port laparoscopic cholecystectomy (TUSPLC) group (study group) or the traditional three-port laparoscopic cholecystectomy (LC) group (control group) based on the surgical approach chosen. The study protocol, including the intraoperative assembly and use of the access port device, was approved by the Medical Ethics Committee of The Affiliated Hospital of Jiangnan University (Ethics Approval No. LS2025057). All patients and their families provided written informed consent after receiving an explanation that the single-port procedure would utilize a non-commercial, intraoperatively assembled access platform using sterile, certified components.

Inclusion Criteria:

1. A discharge diagnosis of benign gallbladder diseases.
2. Onset of cholecystitis within 72 h prior to surgery (if applicable).
3. The patient was in good general condition, with no history of myocardial infarction or cerebral infarction within the past six months and no surgical contraindications, such as cardiac, pulmonary, or renal insufficiency.
4. No history of previous upper abdominal surgery.

Exclusion Criteria:

1. Concomitant bile duct stones, acute pancreatitis, or liver cirrhosis.
2. Requirement for additional intraoperative procedures other than cholecystectomy.
3. Cases in the early phase of the surgical learning curve.
4. Acute cholecystitis graded as III in severity.
5. Inability to complete follow-up evaluations.

### Modified glove-port device fabrication

**Fig. 1:**
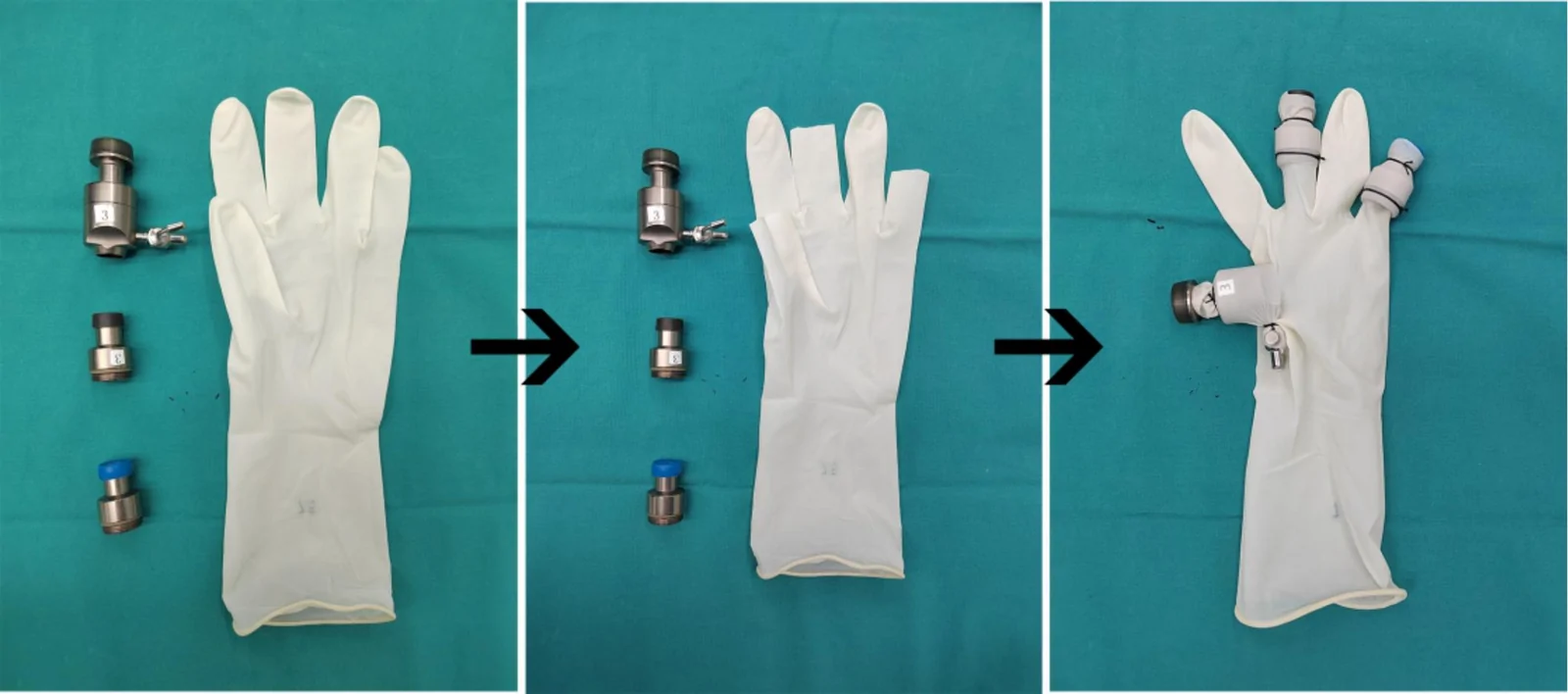
Fabrication of a custom single-port device: A size 7 sterile glove was used. The tips of the thumb, middle finger, and little finger were cut from the glove, and the tips of one 10 mm and two 5 mm trocars (with cannula portions removed) were inserted, and secured with silk suture

A size-7 sterile surgical glove was used. The tips of the thumb, middle finger, and little finger were cut from the glove. The tips of one 10-mm and two 5-mm trocars (with cannula portions removed) were inserted and secured with silk suture. A 10-mm trocar was inserted into the thumb tip as the observation port, a 5-mm trocar was inserted into the little finger tip as the main operating port, and a 5-mm trocar was inserted into the middle finger tip as the auxiliary operating port (Fig. 1). All components used for device assembly (surgical glove, wound protector, and trocars) were individually packaged, sterile, and commercially certified medical devices. The assembly was performed intraoperatively under sterile conditions by the surgical team. This modification builds on the basic glove-port technique reported in previous literature and optimizes trocar fixation and the airtight seal, thereby reducing the “chopstick effect” and intraoperative gas leakage. These features represent the key technical improvements of this modified technique.

### Operative method of the study group

**Fig. 2:**
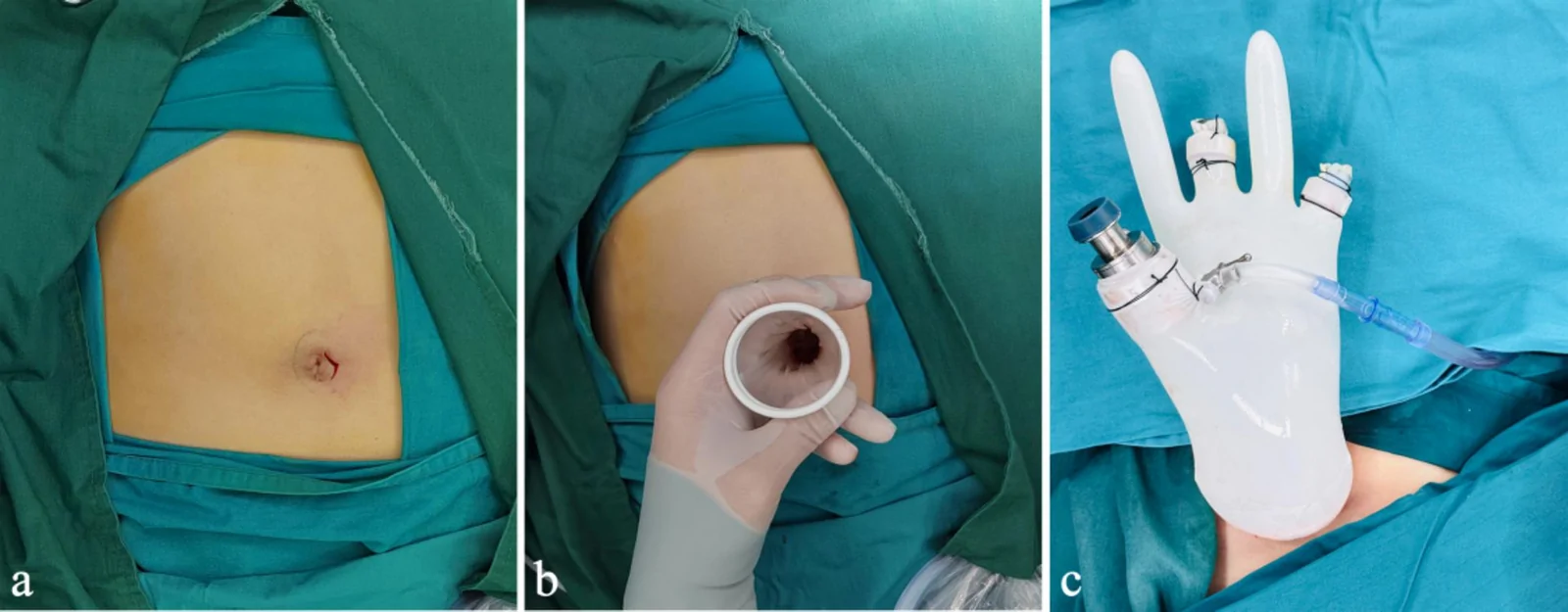
Incision site selection and port placement. **a** A 1.5-cm incision was made along the natural skin crease in the left paraumbilical region. **b **Under direct visualization, a disposable 5-cm wound retractor inner ring was positioned. **c **The outer ring of the custom-made single-port access device was then engaged and cinched tightly to establish an airtight seal

1. Anesthesia and positioning: Each patient received general anesthesia and was placed in a supine position with the legs abducted. Preoperative preparation included thorough cleaning of the umbilical fossa. The primary surgeon stood between the patient’s legs, and the scope holder stood on the patient’s left side.
2. Incision and port insertion: A 1.5-cm incision was made along the natural skin crease in the left paraumbilical region (Fig. 2a). The incision was made through the skin and subcutaneous tissue, followed by a division of the peritoneal layer along the left aspect of the umbilical ring. Under direct visualization, a disposable 5-cm wound retractor inner ring was positioned (Fig. 2b). The outer ring of the custom-made single-port access device was then engaged and cinched tightly to establish an airtight seal (Fig. 2c).

3. Pneumoperitoneum establishment and exploration: Pneumoperitoneum was established with the pressure maintained at 12 mmHg. A 10-mm laparoscopic lens was inserted for exploration, and the patient’s position was adjusted to the reverse Trendelenburg position.
4. Anatomy of the anterior gallbladder triangle: The assistant retracted the gallbladder body or neck with gastric forceps held in the left hand. The primary surgeon dissected the anterior gallbladder triangle with an electrocoagulation hook held in the right hand, isolated the cystic artery, clamped it with a 5-mm Hem-o-Lok clip, and transected the distal segment using an electrocoagulation hook.
5. Anatomy of the posterior gallbladder triangle: The assistant retracted the gallbladder cephalad and laterally to expose and dissect the posterior gallbladder triangle. After dissection, the assistant retracted the gallbladder laterally, and the cystic duct was dissected using a right-angle dissector.
6. Management of the cystic duct: The distal cystic duct was clamped with a 5-mm Hem-o-Lok clip. Half to two-thirds of the diameter of the cystic duct was incised if the cystic duct was thick, the patient was preoperatively diagnosed with sand-like gallstones in the gallbladder, or magnetic resonance cholangiopancreatography (MRCP) indicated stones were in the cystic duct. A dissector was used to squeeze the cystic duct until clear golden-yellow bile flowed out. The proximal cystic duct was then clamped with a 5-mm Hem-o-Lok clip.
7. Gallbladder dissection and removal: The gallbladder was dissected from the gallbladder bed. After hemostasis, the patient was checked for bleeding or bile leakage. The gallbladder was then dragged into the custom-made single port and removed through the umbilical incision.
8. Incision closure: The peritoneum and fascia were sutured to prevent incisional hernia. The incision was sutured internally with absorbable sutures, and no suture removal was required after the operation.

### Operative method of the control group

Patients in the control group underwent traditional three-port LC.

1. Anesthesia and positioning: Each patient was placed in a supine position. After successful general anesthesia, 5-mm, 5-mm, and 10-mm trocars were inserted at the right anterior axillary line, subxiphoid region, and left paraumbilical area, respectively.
2. Surgical procedure: A laparoscope was inserted through the paraumbilical incision, and instruments such as a gallbladder grasper and electrocoagulation hook were inserted through the other two ports. The surgical technique was identical to that of the study group.

### Propensity score matching (PSM)

PSM analysis is increasingly accepted in non-randomized observational studies because it can minimize treatment selection bias. In our study, the two operative techniques were compared using a PSM analysis to minimize patient selection bias and confounding differences between TUSPLC and LC group. Propensity scores were estimated using a multivariable logistic regression model, with surgical approach (TUSPLC vs. LC) as the dependent variable and the following covariates as independent variables: age, sex, BMI, ASA grade, and gallbladder disease type. The model demonstrated acceptable discrimination with a C-statistic of 0.72. Matching was performed using 1:1 nearest-neighbor matching without replacement, in descending order of propensity score, with a caliper width of 0.2 standard deviations of the logit of the propensity score. Covariate balance was assessed using standardized mean differences (SMD); balance was considered adequate when SMD was below 0.1 for all covariates.

### Evaluation of demographic characteristics and outcome measures

Patient medical records, discharge summaries, outpatient/emergency visits, and 1-month telephone follow-up data were retrospectively reviewed. Perioperative data were systematically collected, and relevant laboratory examinations were subsequently assessed. Basic demographic and clinical characteristics collected included sex, age, body mass index (BMI), discharge diagnosis and total hospitalization costs (including examination, surgical, anesthesia, disposable medical consumables, medication, bed, and other fees). The overall physical condition of patients was classified in accordance with the American Society of Anesthesiologists Physical Status Classification System (ASA). Operative time, intraoperative blood loss, postoperative hospital stay, and hospitalization costs, 30-day postoperative complications (including bile leak, intra-abdominal collections, surgical site infection, trocar-site bleeding, readmission, and emergency room visits) were recorded. Abdominal cosmetic satisfaction scores were assessed 1 month postoperatively during outpatient follow-up at our department. The scoring method followed Lee et al. [7], with a 1 ~ 10 scale (10 = optimal satisfaction).

### Statistical analysis

Statistical analyses and PSM were performed using SPSS 22.0 software. Normality of continuous variables was tested using the Shapiro-Wilk test. All continuous variables were analyzed using the t-test (when the variable had a normal distribution in the group) or the nonparametric Wilcoxon rank sum test (when the assumption of normality failed for either group). Categorical variables were presented as rates or proportions, with intergroup comparisons performed using the **χ**^**2**^ test. For the primary analysis of matched pairs, continuous variables were compared using paired t-tests (for normally distributed variables) or Wilcoxon signed-rank tests (for non-normally distributed variables); categorical variables were compared using McNemar’s test or Bowker’s test. Mean differences and 95% confidence intervals (CIs) are reported for all continuous outcomes. Statistical significance was defined as *p* < 0.05. No adjustment for multiple comparisons was made.

## Results

### Preoperative clinical characteristics

During the study period, 173 patients underwent TUSPLC using a custom single-port device (study group). This cohort included 98 males and 75 females, with a mean age of 50.3 ± 18.6 years and BMI of 24.5 ± 3.2 kg/m². Regarding ASA classification in the study group, 112 cases (64.7%) were classified as Grade Ⅰ and 61 cases (35.3%) as Grade Ⅱ. The disease distributions included 54 cases of gallbladder stones with acute cholecystitis (31.2%), 102 with gallbladder stones with chronic cholecystitis (59.0%), 6 with gallbladder polyps (3.5%), 7 with gallbladder stones combined with polyps (4.1%), and 4 with gallbladder stones with atrophic cholecystitis (2.3%). Another 242 patients underwent traditional three-port LC (control group), consisting of 134 males and 108 females with a mean age of 50.7 ± 23.5 years, with a mean BMI of 25.1 ± 3.5 kg/m². The ASA classification was distributed as follows: 159 cases (65.7%) were Grade Ⅰ and 83 cases (34.3%) were Grade Ⅱ. The disease distributions included 89 cases of gallbladder stones with acute cholecystitis (36.8%), 123 cases of gallbladder stones with chronic cholecystitis (50.8%), 9 cases of gallbladder polyps (3.7%), 15 cases of gallbladder stones combined with polyps (6.2%), and 6 cases of gallbladder stones with atrophic cholecystitis (2.5%). After matching, 162 matched pairs were retained, 11 TUSPLC patients (6.4%) and 80 control patients (33.1%) could not be matched and were excluded from the primary analysis. Baseline characteristics did not differ significantly between the two matched cohorts (all *p* > 0.05, Table 1).

**Table 1:** Baseline clinical characteristics of the two groups($\:\overline{\mathrm{x}}$±s/n,%)

Characteristic	Before matching			After matching		
	Study group (*n* = 173)	Control group (*n* = 242)	*p* value	Study group (*n* = 162)	Control group (*n* = 162)	SMD
Sex			0.875			
Male (n)	98	134		87	85	0.024
Female (n)	75	108		75	77	-0.024
Age (y)	50.3 ± 18.6	50.7 ± 23.5	0.847	49.6 ± 18.7	50.3 ± 18.5	0.038
BMI (kg/m²)	24.5 ± 3.2	25.1 ± 3.5	0.071	24.3 ± 2.9	24.6 ± 2.9	0.098
Disease distributions			0.550			
Acute cholecystitis (n, %)	54 (31.2%)	89 (36.8%)		48 (29.7%)	49 (30.2%)	-0.011
Chronic cholecystitis (n, %)	102 (59.0%)	123 (50.8%)		98 (60.5%)	97 (59.9%)	0.012
Gallbladder polyps (n, %)	6 (3.5%)	9 (3.7%)		6 (3.7%)	6 (3.7%)	0.000
Gallbladder stones combined with polyps (n, %)	7 (4.1%)	15 (6.2%)		7 (4.3%)	7 (4.3%)	0.000
Gallbladder stones with atrophic cholecystitis (n, %)	4 (2.3%)	6 (2.5%)		3 (1.9%)	3 (1.9%)	0.000
ASA classification			0.922			
Ⅰ	112(64.7%)	159(65.7%)		109(67.3%)	107(66.0%)	0.028
Ⅱ	61(35.3%)	83(34.3%)		53(32.7%)	55(34.0%)	-0.028

### Operative and postoperative outcomes

Before PSM, all patients in the study group underwent successful laparoscopic surgery, with 3 cases requiring conversion to multi-port approaches. Of these, 2 patients required conversion to three-port LC due to difficult dissection of Calot’s triangle, and 1 patient was converted to two-port LC due to severe gallbladder atrophy. No conversions occurred in the control group. Two of the three converted cases were included in the matched pairs (*n* = 162 per group) and were analyzed as per-protocol outcomes; one converted case was excluded by PSM.

After PSM, mean operative time was 42.0 ± 5.1 min in the study group and 41.2 ± 4.6 min in the control group (mean difference 0.80 min, 95% CI -0.3 to 1.9, *p* = 0.140). Mean intraoperative blood loss was 5.9 ± 1.5 mL and 5.7 ± 1.4 mL respectively (mean difference 0.20 mL, 95% CI − 0.1 to 0.5; *p* = 0.220). Mean postoperative hospital stay was 2.0 ± 0.5 days in the study group and 2.1 ± 0.4 days in the control group (mean difference − 0.10 day, 95% CI − 0.2 to − 0.0; *p* = 0.048). Although the difference in postoperative stay reached statistical significance, the absolute difference was only 0.10 day, which is unlikely to be clinically meaningful. During the 1-month follow-up period, no bile duct injury, postoperative hemorrhage, surgical site infection, bile leakage, readmission, or emergency department visit was recorded in either group. Total hospitalization costs were 14376.2 ± 982.5 CNY in the study group and 14437.3 ± 856.7 CNY in the control group (mean difference − 61.1 CNY, 95% CI − 262.6 to 140.4; *p* = 0.551). One month after the operation, the visual analogue scale (VAS) score was 9.23 ± 0.45 in the study group and 8.52 ± 0.68 in the control group (mean difference 0.71, 95% CI 0.6 to 0.8; *p* < 0.001) (Table 2).

**Table 2:** Comparison of perioperative clinical outcomes between the two groups ($\:\overline{\mathrm{x}}$±s)

	Study group (*n* = 162)	Control group (*n* = 162)	*p* value	Mean difference (95% CI)
Intraoperative blood loss (ml)	5.9 ± 1.5	5.7 ± 1.4	0.220	0.20 (− 0.1 to 0.5)
Operative time (min)	42.0 ± 5.1	41.2 ± 4.6	0.140	0.80 (− 0.3 to 1.9)
Postoperative hospital stay (d)	2.0 ± 0.5	2.1 ± 0.4	0.048	−0.10 (− 0.2 to 0.0)
Hospitalization costs (CNY)	14376.2 ± 982.5	14437.3 ± 856.7	0.551	−61.1 (− 262.6 to 140.4)
Cosmetic satisfaction VAS score	9.23 ± 0.45	8.52 ± 0.68	< 0.001	0.71 (0.6 to 0.8)

## Discussion

### Advantages and limitations of the modified glove-port device using conventional instruments

The emergence of multichannel single-port laparoscopic access devices has significantly advanced single-port laparoscopic surgery [7, 17–19]. However, under China’s DRGs-based medical insurance payment model, the high cost of the devices has limited their routine clinical application in gallbladder surgery [13, 14, 20]. Additionally, when conventional trocar instruments are used for TUSPLC, the “chopstick effect” is pronounced, and air leakage frequently occurs, increasing the difficulty and risk of surgery [19, 21].

To overcome these difficulties, we optimized the construction of a glove-port access device using conventional steel trocars. This improvement not only effectively reduced the “chopstick effect” but also prevented air leakage during surgery, significantly enhancing the smoothness and safety of the operation. Moreover, this modified glove-port device reduces the cost of single-port special trocar consumables and is easy to assemble, making it suitable for routine TUSPLC in resource-limited primary-level hospitals under China’s DRGs payment model. In this study, total hospitalization costs did not differ significantly between the TUSPLC and conventional LC groups. This lack of difference may be attributable to the fact that neither group used disposable commercial multichannel ports; the control group also employed conventional reusable trocars. The term ‘low-cost’ in this context refers specifically to the elimination of expensive commercial multichannel single-port devices (typically costing 1,200-2,500 CNY per unit). While the overall hospitalization costs were comparable, the avoidance of disposable single-port port expenses makes this technique economically advantageous, particularly in resource-limited settings under China’s DRG payment system. However, this study does not constitute a formal cost-effectiveness analysis, and such an analysis would be required to draw definitive conclusions about economic superiority.

Furthermore, the modified glove-port device is equipped with a wound protector, which effectively protects the umbilical incision from contamination by bile, pus, and other substances during surgery, thereby potentially reducing the incidence of postoperative incision infection and incisional hernia [22]. In this study, no surgical site infections were observed in either group during the 1-month follow-up. While this finding supports the theoretical protective effect of wound retractors, comparable infection rates between the two groups prevent causal attribution to the glove-port device. Prospective studies with larger cohorts and prolonged follow-up are required to confirm this benefit. The flexibility of the single-port channel also facilitates gallbladder extraction, reducing secondary trauma to the umbilical incision, further alleviating postoperative pain, and promoting postoperative recovery.

In the present series, 3 out of 173 patients (1.7%) required conversion to multi-port approaches. Two conversions were due to difficult Calot’s triangle anatomy, and one was due to severe gallbladder atrophy. These cases highlight the practical limitations of the TUSPLC technique in patients with complex anatomy or atrophic gallbladders. Surgeons should maintain a low threshold for conversion when encountering difficult dissection, as patient safety should always be prioritized over cosmetic outcomes.

### TUSPLC exposure techniques

**Fig. 3:**
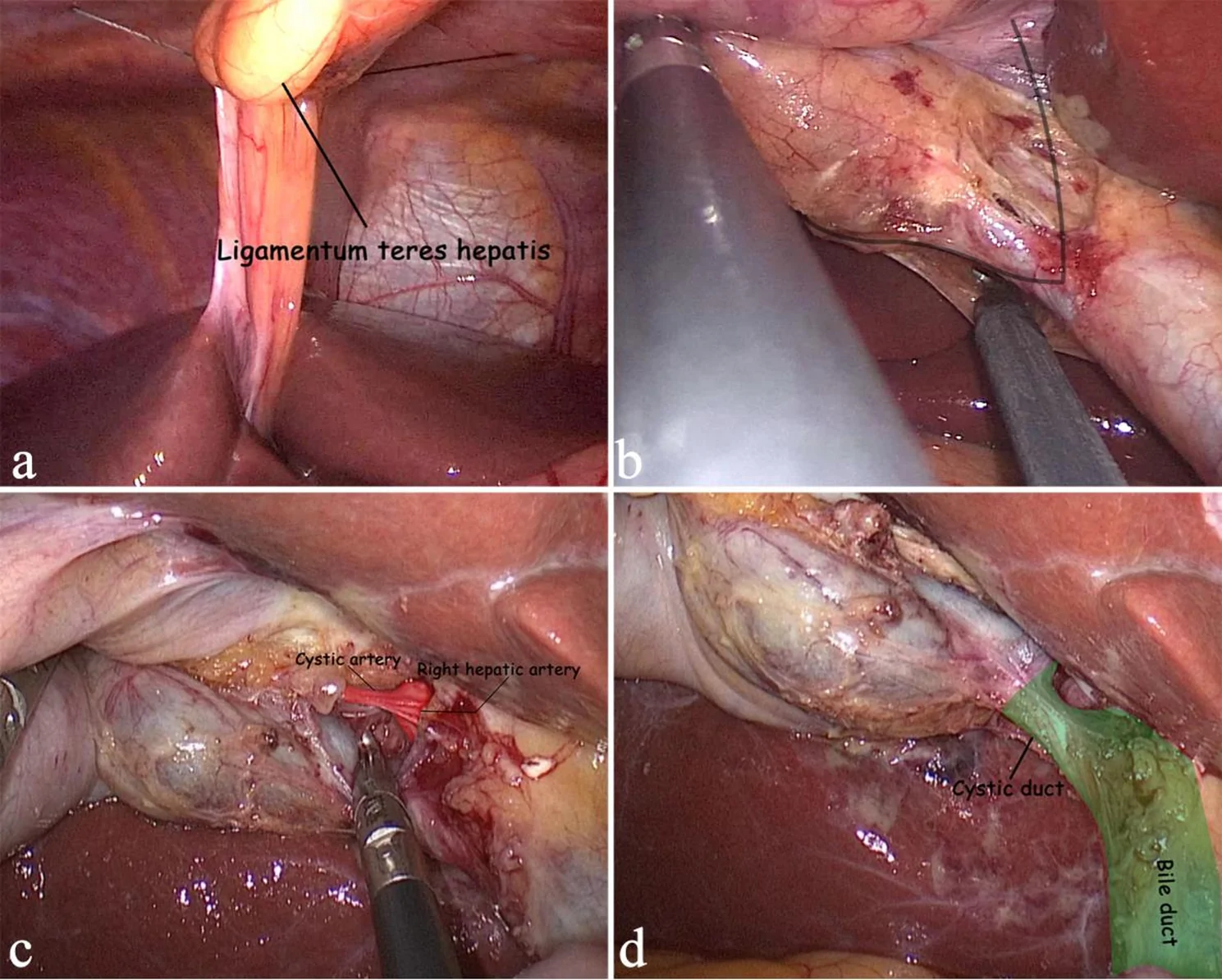
TUSPLC exposure techniques suspension of the ligamentum teres hepatis (**a**). V-shaped approach to dissect the gallbladder’s anterior and posterior triangles (**b**). Complete dissection and clear identification of the cystic artery and right hepatic artery (**c**). Clear visualization of calot’s triangle (**d**)

In this study, all patients were placed in the supine position with legs abducted, with the primary surgeon between the legs and the assistant on the left. A purse-string suture was used to maximally elevate the ligamentum teres hepatis cephalad and to the left (Fig. 3a). This technique not only preserves the integrity of the patient’s upper abdominal skin but also effectively exposes the anterior gallbladder triangle. By maintaining tension and retracting the gallbladder laterally and cephalad, the assistant can clearly visualize the anterior gallbladder triangle, providing an optimal surgical field.

In our TUSPLC technique, we adopted a V-shaped approach to sequentially incise the serosal layers of the anterior and posterior gallbladder triangles (Fig. 3b). In the anterior gallbladder triangle, the surgeon dissected and skeletonized the cystic artery (Fig. 3c). Given that the cystic artery most commonly originates from the right hepatic artery, special care was taken to identify the right hepatic artery to avoid iatrogenic injury. Prioritizing dissection of the cystic artery helps minimize intraoperative bleeding, whereas clear dissection of the anterior gallbladder triangle effectively minimizes the risk of bile duct injury. Additionally, after cystic artery management, the gallbladder was more easily retracted laterally and cephalad, facilitating further dissection of Calot’s triangle and full visualization of the relationships among the three ducts (cystic duct, common hepatic duct, and common bile duct) (Fig. 3d), thereby enhancing surgical safety and efficiency.

### Management of cystic duct calculi

According to a study by Mahmud et al. [23], the incidence of cystic duct calculi during cholecystectomy is as high as 12.3%. Residual cystic duct calculi are associated with postoperative complications and may increase the long-term risk of cholangiocarcinoma [24]. Therefore, meticulous management of cystic duct calculi during cholecystectomy is highly important.

To reduce the risk of postoperative residual cystic duct calculi, we performed comprehensive preoperative imaging evaluations for all patients. For patients without significant contraindications, MRCP and ultrasonography were routinely performed to identify cystic duct calculi and biliary ductal anatomical variations. Adequate preoperative assessment provided critical information for surgical planning.

**Fig. 4:**
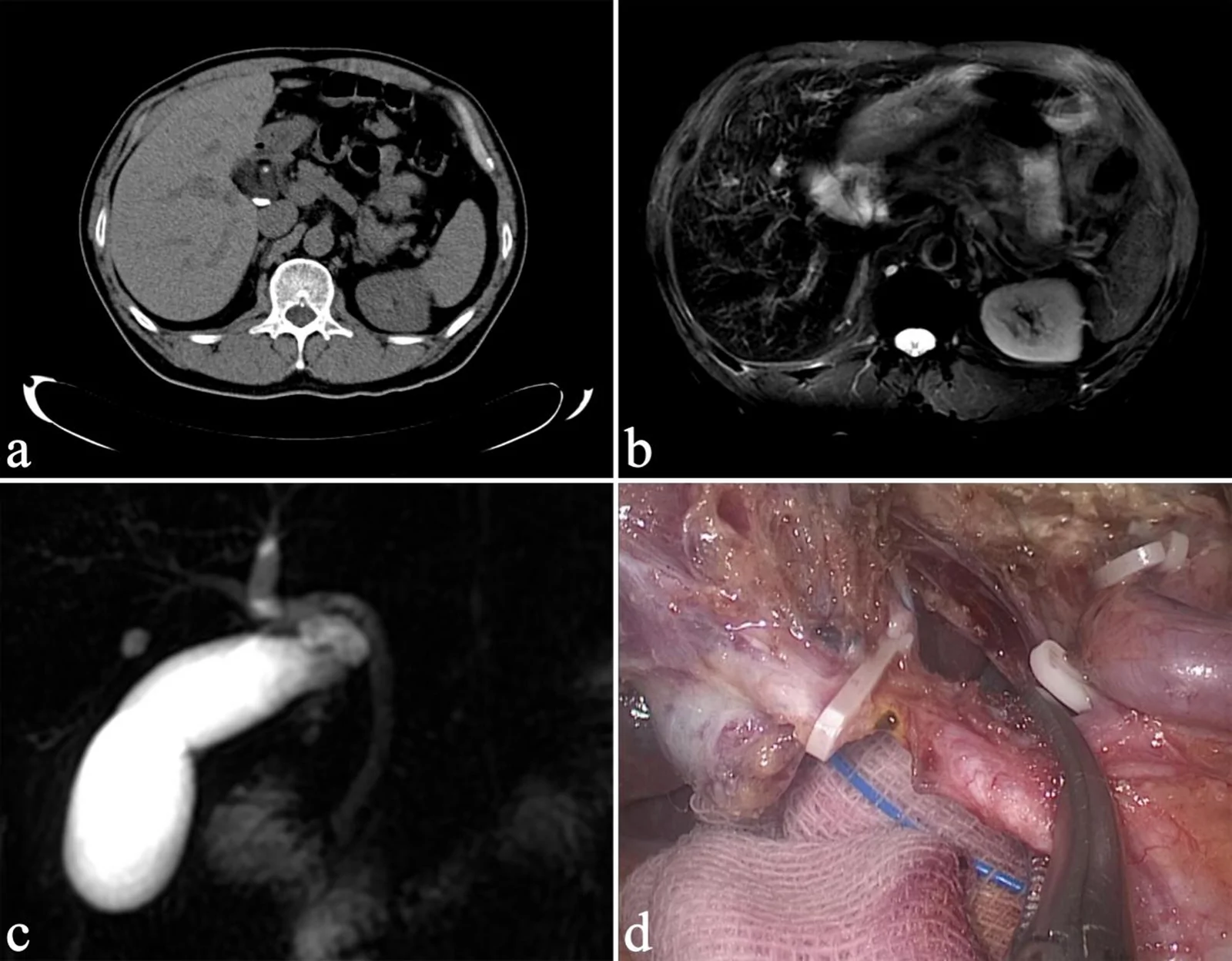
Management of cystic duct stones preoperative imaging for the diagnosis of cystic duct stones (**a-c**). Intraoperative techniques for managing cystic duct stones (**d**)

For patients with cystic duct calculi identified on preoperative imaging or those with a dilated cystic duct and sand-like gallbladder calculi detected intraoperatively (Fig. 4a-c), we employed the following management strategy. First, the cystic duct was skeletonized to ensure clear visualization. The distal cystic duct was then clipped with a 5-mm Hem-o-Lok clip to prevent stone migration into the common bile duct. After the cystic duct was incised, gentle compression of the proximal cystic duct with a dissector was performed to extrude the calculi until clear golden bile drained from the proximal duct (Fig. 4d). This technique effectively removes cystic duct stones and reduces the risk of residual calculi and associated complications.

By using these techniques, no residual cystic duct calculi or related complications were observed in this study, ensuring long-term postoperative outcomes for patients. This strategy offers a practical solution for managing cystic duct calculi in resource-limited settings.

### Limitations of the study

This study has several limitations that should be acknowledged. Firstly, this is a retrospective, non-randomized and single-center study. The surgical approach was selected after patient counseling and according to patient preference, and cases from the early learning curve were excluded. Thus, selection bias cannot be fully excluded, and the results reflect outcomes in experienced hands. We performed propensity score matching to minimize baseline differences, but residual confounding from unmeasured variables may persist. Thus, the results reflect outcomes in experienced hands and may not be generalizable to all clinical settings. The exclusion of early learning-curve cases may have introduced selection bias by reflecting only outcomes after technical proficiency was achieved; however, this exclusion was intended to focus the analysis on the performance of the established technique in routine practice. Secondly, postoperative follow-up was limited to only 1 month after surgery, which is inadequate to assess long-term adverse events including port-site hernia, chronic abdominal pain, delayed incision complications and long-term biliary complications. Although no complications were recorded in medical records within 30 days, retrospective data collection carries risks of incomplete medical documentation and underreporting of mild adverse events. The sample size of the current cohort lacks sufficient statistical power to analyze rare complications such as bile duct injury and port-site hernia; zero observed adverse events in this short-term follow-up does not indicate complete elimination of these long-term risks. These complications are rare events that require much larger sample sizes and longer follow-up periods to assess adequately.Therefore, these results support short-term technical feasibility but do not establish long-term safety. Third, the 1-month follow-up period was too short to assess long-term outcomes, particularly port-site hernia, a key concern after transumbilical surgery. Accordingly, this study only suggests the short-term feasibility of the procedure; long-term safety requires further verification with extended follow-up (≥ 6 months) and larger studies. Fourth, cosmetic satisfaction was only assessed at 1 month postoperatively using a non-validated scale without blinded evaluation. Furthermore, as patients received preoperative counseling about the cosmetic advantages of TUSPLC before choosing their surgical approach, expectation bias may have contributed to the higher satisfaction scores in the study group. Thus, this finding reflects short-term patient-reported satisfaction and cannot be interpreted as evidence of superior objective cosmetic outcomes. Fifth, given the multiple outcomes examined, p-values are reported without formal multiplicity adjustment, and results should be interpreted with appropriate caution.

## Conclusions

**Fig. 5:**
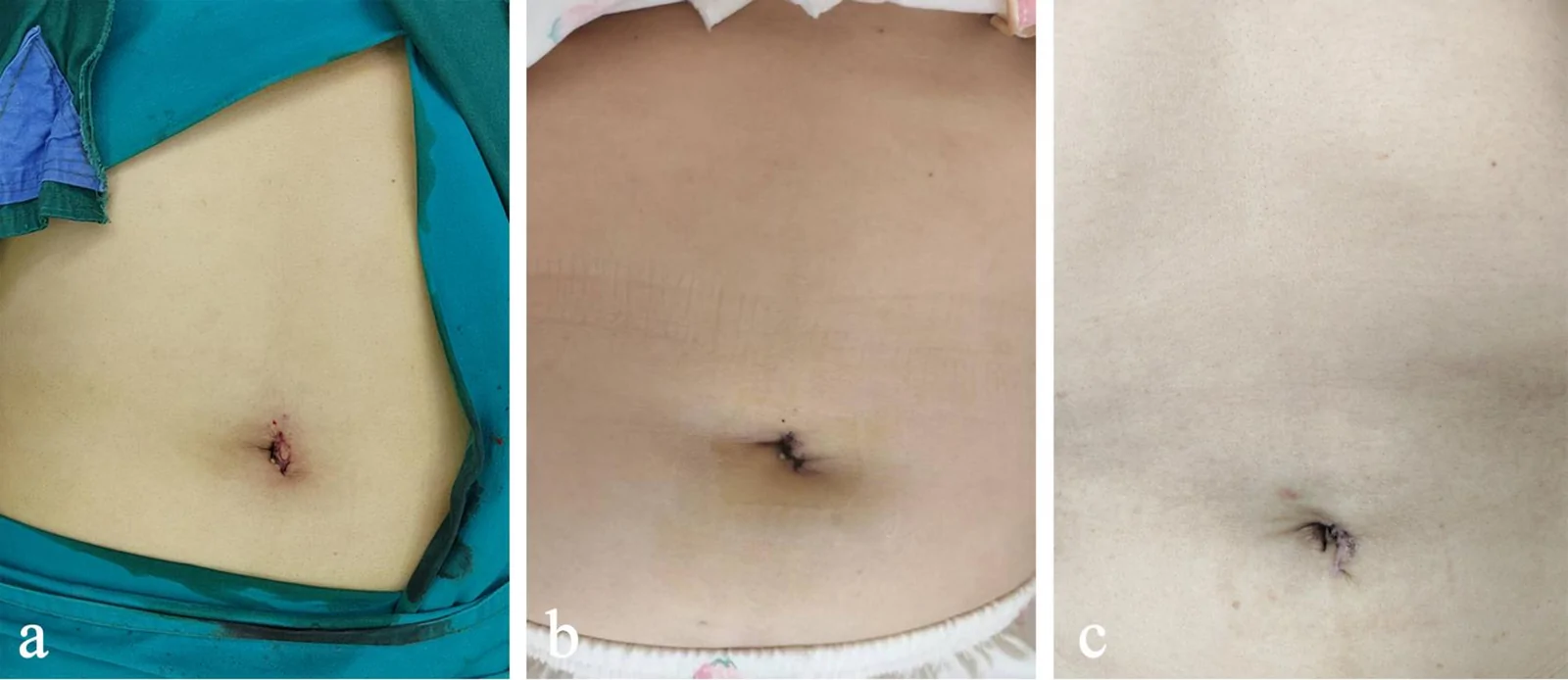
Healing status of the patient’s incision after postoperative cosmetic closure (**a**). One week postoperatively, the incision healing status was evaluated (**b**). Two weeks postoperatively, the incision healing status was evaluated (**c**)

Within the limitations of this single-center retrospective study, TUSPLC using a modified glove-port device and conventional instruments appears technically feasible with acceptable short-term perioperative outcomes. The modified technique showed no statistically significant differences in operative time, blood loss, or hospitalization costs compared with conventional three-port LC, although postoperative hospital stay was statistically shorter by 0.10 day, a difference of uncertain clinical significance. A patient-reported cosmetic satisfaction advantage was observed at 1 month (Fig. 5). However, due to the retrospective, single-center design, short (1-month) follow-up period, exclusion of early learning-curve and conversion cases, and lack of statistical power for rare events, these findings should be considered preliminary. Overall, the modified technique provides a practical strategy for implementing TUSPLC in resource-limited settings, particularly primary hospitals under China’s DRGs payment model. Further prospective, randomized, multicenter studies with extended follow-up (≥ 6 months) and validated objective outcome assessments are essential to validate the long-term safety and generalizability of this technique.

## Data Availability

The datasets generated and/or analyzed during the study are not publicly available because we did not receive permission for them to be accessible to researchers from outside organizations. However, they will be available from the corresponding author upon reasonable request after obtaining permission from the Medical Ethics Committee of The Affiliated Hospital of Jiangnan University.
